# Drought stress tolerance and metabolomics of *Medicago sativa* induced by *Bacillus amyloliquefaciens* DGL1

**DOI:** 10.3389/fpls.2024.1378707

**Published:** 2024-05-13

**Authors:** Xue Yang, Yongli Xie, Youming Qiao, Feifei Chang, Tian Wang, Junxi Li, Lingling Wu, Chen Li, Ying Gao

**Affiliations:** ^1^ College of Agriculture and Animal Husbandry, Qinghai University, Xining, China; ^2^ Key Laboratory of Use of Forage Germplasm Resources on Tibetan Plateau of Qinghai Province, Qinghai University, Xining, China; ^3^ State Key Laboratory of Plateau Ecology and Agriculture of Qinghai University, Xining, Qinghai, China; ^4^ Xining Forestry Scientific Research Institute, Xining, Qinghai, China

**Keywords:** *Bacillus amylolyticus*, alfalfa, drought stress, antioxidant enzymes, growth promotion, metabolomics

## Abstract

**Introduction:**

This study used *Bacillus amyloliquefaciens* DGL1 isolated from the arid sandy land of the Qinghai–Tibetan Plateau as the research strain and investigated the effects of DGL1 on the biomass, physiology, and metabolites of *Medicago sativa* under different intensities of drought stress to provide a high-quality bacterial source and a theoretical basis for the research and development of biological fertilizer suitable for arid areas.

**Methods:**

The exopolysaccharides (EPS), 1-Aminocyclopropane-1-carboxylate deaminase (ACC), and phosphorus solubilizing capacity of DGL1 were determined. The effects of a DGL1 suspension on alfalfa biomass, physiological indexes, degree of peroxidation of cell membranes, and activity of antioxidant enzymes were determined after irrigating roots under drought stress. The effects on soil physicochemical properties were also evaluated, and metabolomics analysis was performed to explore the effect of DGL1 on the metabolites of alfalfa under drought stress.

**Results:**

Strain DGL1 produced extracellular polysaccharide EPS and ACC deaminase and was capable of phosphorus solubilization. Treatment with DGL1 increased the biomass of alfalfa under different degrees of drought stress, significantly increased the activities of alfalfa antioxidant enzymes *Super Oxide Dismutase* (SOD), Peroxidase (POD), and catalase (CAT), reduced the content of MDA and H_2_O_2_, and increased the content of quick-acting phosphorus, quick-acting potassium, ammonium nitrogen, and nitrate nitrogen in the soil, thus improving soil fertility. Through metabolomics analysis, DGL1 was shown to affect amino acid metabolic pathways, such as arginine, leucine, glutamate, and tyrosine, as well as the levels of energy-providing polysaccharides and lipids, in alfalfa under 15% PEG-6000 drought stress, enhancing alfalfa’s capacity to resist drought stress.

**Discussion:**

Strain DGL1 enhances the drought suitability of alfalfa and has the potential for dryland development as a biological agent.

## 1 Introduction

Alfalfa, which has a high grass yield, strong resistance, and good palatability, is rich in proteins, amino acids, and a variety of vitamins, in addition to being a high-quality legume forage grass consumed by livestock (Carter and Sheaffer, 1983). Alfalfa has a well-developed root system that can fix free nitrogen in the air with the help of rhizobacteria to improve soil quality, crucial to the agriculture and animal husbandry industries, and therefore being planted all over the world (Radović et al., 2009). Although alfalfa is highly drought tolerant, it requires a large amount of water for growth; thus, cultivation in arid and semi-arid areas is overly dependent on irrigation, and the growth process is susceptible to drought stress (Attram et al., 2016). The growth indicators of alfalfa, such as stem length, stem thickness, number of tillers, and leaf area, decrease after being subjected to drought stress (He et al., 2022). Alfalfa growth and physiological characteristics can also be limited. Irigoyen found that drought stress reduced the photosynthetic rate of alfalfa, significantly increased the MDA and H_2_O_2_ content, and inhibited peroxidase synthesis (Irigoyen et al., 1992). In addition, the nutritional value of alfalfa decreases after a period of drought (Liu et al., 2018).

To promote sustainable plant production in adverse environments, the use of beneficial microorganisms has become an effective means of mitigating abiotic stress. Tolerant microorganisms increase the adaptive capacity to abiotic stress by enhancing the antioxidant potential of the plant, improving nutrient acquisition, preventing fungal or bacterial diseases, modulating phytohormones, and inducing systemic resistance in a variety of ways, thus increasing plant biomass and crop yield (Basu et al., 2021). Rashid’s screening of rhizospheric *Bacillus* in arid and semi-arid regions and under drought stress on wheat seed germination and seedling growth showed that inoculation with *Bacillus megaterium* increased wheat germination and biomass and enhanced the drought tolerance of wheat (Rashid et al., 2022). Ethylene produced by plants under biotic or abiotic stress has been reported to cause tissue damage and interfere with normal plant growth and development. Interestingly, ACC deaminase produced by plant growth-promoting rhizobacteria (PGPR) cleaves the ethylene precursor substance, ACC, produced by the host plant into α-Ketobutyric acid and ammonia, thereby mitigating the inhibitory effect of excess ethylene on the growth of the plant body (Murali et al., 2021). Sarapat used genetic engineering to enhance the production of ACC deaminase activity using *Bradyrhizobium* strains, and the modified strains improved symbiosis with plants and drought tolerance (Sarapat et al., 2020). In addition, *Bacillus* improves the drought tolerance of host plants by secreting antioxidant enzymes that help plants scavenge oxygen radicals (Saikia et al., 2018). Inoculation with *Bacillus* has been reported to significantly increase the activities of the antioxidant enzymes SOD, CAT, and POD in wheat, suggesting that microbial inoculation has the potential to alleviate water stress in wheat (Sood et al., 2020). Moreover, EPS produced by *Bacillus* can maintain stable soil and water binding, which increases the inter-root available water for plants, promotes soil water-holding capacity and fertility, and enhances microbial tolerance under drought stress (Hale et al., 2021). *Bacillus* can also convert insoluble phosphate in the soil into effective phosphorus that can be absorbed and utilized by plants, improving the utilization rate of phosphorus in the soil and thus increasing the phosphorus nutrition of plants (Zhang et al., 2023). Maria reported that the phosphorus absorption rate of cucumber increased by 40% after inoculation with *Bacillus subtilis* QST713 (Ana María and Delgado, 2016). With a fast reproduction rate, high resistance and metabolism ability, and wide distribution range, *Bacillus* is a transforming factor for insoluble compounds, such as nitrogen, phosphorus, and potassium, in the soil environment, which improves soil fertility, indirectly provides more nutrients to the host plant, and helps the plant to adapt to an adverse environment (Shen et al., 2023).

Few studies have been reported on the application of isolated indigenous *Bacillu*s screened on the Tibetan Plateau for the growth of pasture grasses under drought stress. In the present study, we used *Bacillus amylolyticus* DGL1 isolated from the rhizosphere of *Nitraria tangutorum* in the arid sandy area of Dagler, Haixi Prefecture, Qinghai Province, China (altitude 3010 m), as the object of study. We studied the effect of DGL1 on the growth indexes and physiological characteristics of alfalfa under drought stress and used metabolomics to explore the induction of differential metabolites by *Bacillus* DGL1 in alfalfa. The results of this study provide new ideas and a scientific basis for the improvement of the adaptive ability of alfalfa under drought adversity using *Bacillus*.

## 2 Materials and methods

### 2.2 Determination of the biological activity of strain DGL1

#### 2.2.1 EPS production capacity

After overnight incubation of strain DGL1 in LB liquid medium (200 rpm, 37°C), strain DGL1 was used to inoculate EPS special medium (2% yeast extract, 1.5% K_2_HPO_4_, 0.02% MgSO_4_, 0.0015% MnSO_4_, 0.0015% FeSO_4_, 0.003% CaCl_2_, 0.0015% NaCl, 1.5% agar, 10% sucrose, pH 7.5) and incubated for 2 d. EPS production was determined by the appearance of mucus on the surface of the medium (Jian-Feng, 2009).

#### 2.2.2 ACC deaminase-producing ability

Strain DGL1 was cultured in a nitrogen-free liquid medium (200 rpm, 37°C) for 1 d, and 100 μL of the above bacterial solution was aspirated and used to inoculate DF basic medium for 1 d. The strain was then transferred to 0.5 M DF basic medium (ADF) with ACC as the only nitrogen source for 2 d (200 rpm, 30°C). The strain, if grown normally in ADF medium, had the ability to produce ACC deaminase (Murali et al., 2021).

The ACC deaminase (ACCD) enzyme immunoassay was used to determine the amount of ACC deaminase produced by DGL1. Blank wells, standard wells, and wells of the samples to be tested were set up on an enzyme-coated plate. In the enzyme-coated plate, 50 μL of standard was added accurately, and 40 μL of sample diluent was added to the sample wells to be tested. Then, 10 μL of the sample to be tested was added, incubated for 30 min, and washed five times with a washing solution. A volume of 50 μL of enzyme-labeling reagent was added, and the plates were incubated for 30 min and washed five times. The colorant and termination solutions were added, and the absorbance was measured at 450 nm.

#### 2.2.3 Phosphorus solubilization capacity

Strain DGL1 was used to inoculate Monkina medium plates and cultured at a constant temperature of 28°C for 10 d. The size of the phosphorus-solubilizing circle of strain DGL1 was determined and the phosphorus-solubilizing ability of the strain was initially determined by the ratio of the diameter of the phosphorus-solubilizing circle (D) to the diameter of the colony (d) (D/d).

The ability of the strain to detoxify phosphorus was determined using vanadium-molybdenum colorimetry. Strain DGL1 was used to inoculate LB liquid medium, incubated overnight at 28°C, and centrifuged to collect the bacteria. The bacteria were diluted with sterile water to make a bacterial suspension, and 100 μL of DGL1 bacterial suspension was used to inoculate the fermentation medium, incubated for 3 d (30°C, 160 r/min), and centrifuged (12,000 g). Then, 1 mL of supernatant was added to 1 mL of sodium phytate solution at a concentration of 8.4 g/L. The OD_415_ nm was measured after holding it at 37°C for 30 min (Mohamed et al., 2018).

### 2.3 Determination of the effect of DGL1 on physiological indicators of alfalfa seedlings under drought stress

#### 2.3.1 Alfalfa biomass

Alfalfa grass seeds with intact seed coats were selected and placed in a 20% sodium hypochlorite solution for 10 min to sterilize the seeds. After rinsing with sterile water 5 times, the treated seeds (25 plants/pot) were sown in cavity pots (V_nutrient soil_: V_natural soil_ = 2:1) and cultivated in a light incubator (25°C, photoperiod 16 h/8 h). When the plant height reached about 4 cm, drought stress was simulated using 0, 5, 10, 15, 20, and 25% PEG-6000 (the corresponding water potential was 0, -0.05, -0.15, -0.30, -0.50and -0.77MPa, respectively) in a control (tap water, CK) and bacterial solution DGL1 treatment group (bacterial suspension DGL1 irrigated roots, DGL1) with three replications. After 45 d of growth, plant samples were collected to determine plant height, fresh weight, root length, root volume, root surface area, root tip number, and root average diameter using the Root Analysis System.

#### 2.3.2 Degree of peroxidation in alfalfa cell membrane systems

The H_2_O_2_ content was determined using a CheKine™ Hydrogen Peroxide Assay Kit. Alfalfa leaves (0.1 g) were added to 1 mL of pre-cooled assay buffer (1×), ground thoroughly, and centrifuged at 12,000 g for 10 min at 4°C. The supernatant was collected as the sample to be tested. The procedure was performed according to the manufacturer’s instructions, and the H_2_O_2_ content was calculated.

The MDA content was determined using a Solarbio Malondialdehyde Content Detection Kit. Briefly, 0.1 g of alfalfa leaves was added to 1 mL of extraction solution, ground thoroughly, and centrifuged at 8000 g for 10 min at 4°C. The supernatant was placed on ice to be measured. The procedure was performed following the manufacturer’s instructions, and the MDA content was calculated.

#### 2.3.3 Alfalfa antioxidant enzyme activity

SOD activity was determined using a CheKine™ SOD Activity Assay Kit. Briefly, 0.1 g of alfalfa leaves was added to 1 mL of pre-cooled 1× lysis buffer and ground. After thorough grinding with a glass homogenizer, the sample was centrifuged for 5 min at 12,000 g at 4°C. The supernatant was collected as the sample to be tested. The procedure was performed according to the instruction manual, and the SOD content was calculated.

POD activity was determined using a CheKine™ POD Activity Assay Kit. The alfalfa leaves were washed with cold PBS. The water was removed, and the leaves were cut into pieces. A 0.1 g leaf sample was added to 1 mL of pre-cooled extraction buffer and centrifuged at 8000 g for 10 min at 4°C. The supernatant was collected for measurement. The procedure was performed following the manufacturer’s instructions, and the POD content was calculated.

CAT activity was determined using a Solarbio CAT Activity Assay Kit. The ratio of the mass of fresh alfalfa tissue (g) to the volume of the extraction solution (mL) was 1:5–10. The mixture was homogenized in an ice bath and centrifuged at 8000 g for 10 min at 4°C. The supernatant was collected and placed on ice to be measured. The CAT content was determined according to the manufacturer’s instructions and calculated.

### 2.4 Determination of the soil amelioration effect of DGL1 under drought stress

#### 2.4.1 Soil quick-acting phosphorus

After treatment following section 2.3.1, 1.0 g of air-dried soil was added to 50 mL of 0.5 mol/L NaHCO_3_ solution, shaken for 30 min, filtered, and aspirated. A volume of 10 mL of filtrate was added to 35 mL of distilled water and 5 mL of molybdenum-antimony reagent, shaken well, and left to stand for 30 min. A blank group was used as the control, and a UV spectrophotometer was used to colorimetrically compare the results at a wavelength of 700 nm (Bian and Ma, 2018) and to plot the standard curve. The phosphorus content was calculated from the standard curve, and the quick-acting phosphorus content was calculated using the following formula:


$$\text{effective phosphorus content }=\frac{\rho \times V\times t}{m\times k}$$

where *P* is the concentration of phosphorus on the standard curve (μg/mL), V is the volume of the color-developing solution, t is the ratio of the total volume to the volume of the color-developing solution, and m is the mass of air-dried soil.

#### 2.4.2 Soil quick-acting potassium

Soil quick potash was determined using the flame photometer method. NH_4_Ac extractant was mixed with soil at a ratio of 10:1, shaken, and leached for 30 min. Dry filtration and the filtrate were used to determine the potassium content on a flame photometer (Moody and Bell, 2006).

#### 2.4.3 Soil ammonium nitrogen and nitrate nitrogen

A 2.00 g soil sample was placed in a 50 mL glass extraction flask, and 20 mL of 0.01 mol/L calcium chloride leachate was added, extracted by shaking for 60 min, and dry filtered. To determine the ammonium nitrogen and nitrate nitrogen content, 5–10 mL of filtrate was collected and used in an upper flow injection analyzer (Ghafoor et al., 2021).

#### 2.4.4 Data analysis

The data were statistically analyzed using the SPSS program. Mean values were estimated based on Duncan’s test (*P*< 0.05), and indicators were expressed as the mean ± standard deviation (x± SD).

### 2.5 Determination of the effect of DGL1 on the metabolome of alfalfa under drought stress

#### 2.5.1 Interaction of alfalfa with DGL1 under drought stress

Grass seeds of alfalfa with intact seed coats were selected, sterilized, and sown in cavity pots (8 cm in diameter × 10 cm in height) and placed in a light incubator (temperature 25°C, photoperiod 16 h/8 h), Alfalfa seedlings cultured for 30 d were removed from cavity pots, and the roots were washed several times with sterile water. Filter paper was used to absorb the water from the roots, and they were divided into four groups: a: alfalfa roots completely immersed in sterile water (CK); b: alfalfa roots immersed in DGL1 bacterial suspension with a cell concentration of 1 × 10^6^ cfu·mL^−1^ (DG); c: alfalfa roots immersed in 15% PEG-6000 solution as a drought-treated group; and d: alfalfa roots immersed in DGL1 and 15% PEG-6000 solution as a drought + strain DGL1 treatment group (DGd). After 4 h of treatment, the leaves were rapidly cut off and frozen in liquid nitrogen as samples to be tested.

#### 2.5.2 Sample preparation

Alfalfa leaf samples (1 g) were collected from each of the four treatment groups in 2 mL centrifuge tubes, and grinding beads and extraction solution were added for metabolite extraction. The extract was ground for 6 min in a frozen tissue grinder and sonicated for 30 min at a low temperature. The samples were left at −20°C for 30 min and centrifuged for 15 min, and the supernatant was transferred to sample vials for LC-MS/MS analysis.

#### 2.5.3 Data preprocessing and library searching

Raw data obtained after onboarding were imported into the metabolomics processing software Progenesis QI (Waters Corporation, Milford, USA) for baseline filtering, peak identification, integration, retention time correction, peak alignment, and generation of a data matrix of retention times, mass-to-charge ratios, and peak intensities.

#### 2.5.4 Analysis of differential metabolites

Principal component analysis (PCA) and orthogonal least partial squares discriminant analysis (OPLS-DA) were performed on the data matrices using R software package ropls (Version 1.6.2) with Student’s *t* test and multiplicity of difference analysis. Metabolites with VIP > 1 and *P*< 0.05 were identified as differential metabolites. Differential metabolites and metabolic pathways were annotated using the Kyoto Encyclopedia of Genes and Genomes (KEGG) database.

### 2.6 DGL1 gene annotation analysis

The whole genome sequence of DGL1 was uploaded to NCBI under accession number CP05539. The bacterial genome sequence was rapidly annotated using Gene Ontology (GO), KEGG, and Clusters of Orthologous Groups of proteins (COG), and the corresponding annotation information was obtained.

## 3 Results and analysis

### 3.1 Bioactivity of strain DGL1

#### 3.1.1 EPS production capacity

**Figure 1:**
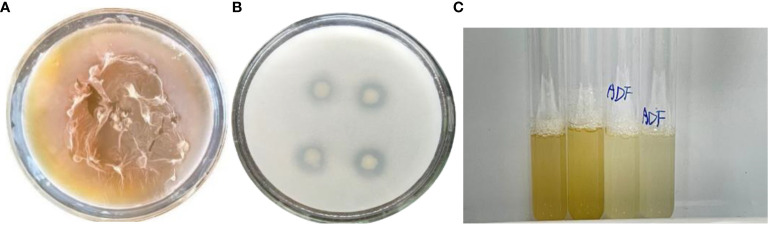
Determination of DGL1 biological activity. **(A)** Determination of EPS-producing activity, the EPS medium produced a large amount of mucus after 12 h of incubation; **(B)** Capacity of PGPR strains, obvious phosphorus-solubilizing circle was produced after 6 d of incubation; **(C)** Growth of DGL1 in DF and ADF media, Strain DGL1 grew in DF basic medium with ACC as the sole nitrogen source.

Strain DGL1 was cultured on a special EPS medium, and obvious mucus rings appeared after 4 h. The medium produced a large amount of mucus after 12 h of incubation, and it was initially determined that strain DGL1 had EPS-producing activity (**Figure 1A**). By analyzing the genome of strain DGL1 (accession number: CP05539), DGL1 was found to have extracellular polysaccharide synthesis-related coding genes, including the α-glucosidase-encoding gene *malL*, the α-galactosidase-encoding gene *melA*, the α-6-phosphoglucosidase-encoding gene *bglA*, the β-xylosidase-encoding gene *xynD*, the endocytosis of β-1,4-xylanase-encoding gene *srfJ*, and the chitosanase-encoding gene *csn*.

#### 3.1.2 Phosphorus-solubilizing ability

To determine the phosphorus-solubilizing ability of the strain, DGL1 was used to inoculate Monkina medium plates, and an obvious phosphorus-solubilizing circle was produced after 6 d of incubation (**Figure 1B**). The diameter of the phosphorus-solubilizing circle was 2.31 cm, and the diameter of the colony was 1.13 cm, with a ratio of 2.044. The phosphorus-solubilizing ability of the strain was quantified by vanadium and molybdenum colorimetry, and the standard curve was y = 0.7925x + 0.0071 (R^2 ^= 0.9993). The amount of phosphorus solubilized by DGL1 was 44.47 ± 2.19 mg/L, indicating that DGL1 had a strong phosphorus-solubilizing capacity.

#### 3.1.3 ACC deaminase-producing capacity

Strain DGL1 grew in DF basic medium with ACC as the sole nitrogen source, initially indicating that DGL1 possessed ACC deaminase activity (**Figure 1C**). The ACC deaminase (ACCD) enzyme immunoassay kit was used for quantitative analysis, and the standard curve of ACC deaminase was measured as y = 0.0176x + 0.0056. Based on the absorbance, the ACC deaminase activity of DGL1 was 25.25 ng/L.

### 3.2 Effect of DGL1 on physiological indicators of alfalfa seedlings under drought stress

#### 3.2.1 Changes in alfalfa biomass

**Figure 2:**
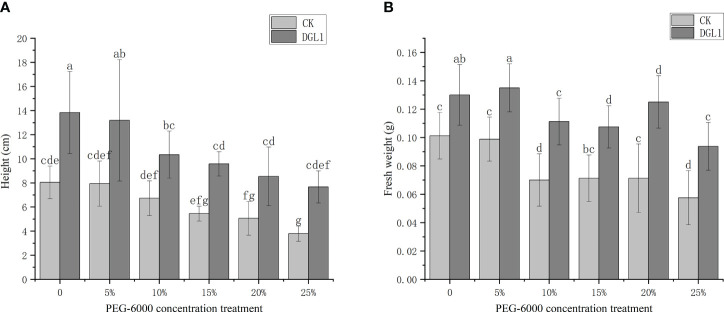
Effect of strain DGL1 on the height and fresh weight of *Medicago sativa* under drought stress: **(A)** plant height; **(B)** plant fresh weight. CK (water-treated group) DGL1 (group in which the roots were irrigated with a mycorrhizal suspension of DGL1); horizontal coordinates indicate 0, 5, 10, 15, 20, and 25% PEG-6000 drought treatments, different letters denote significant differences (P< 0.05) between various treatments based on one-way analysis of variance and Duncan’s test.

As the degree of drought stress increased, the plant height and fresh weight of alfalfa in the control group decreased. Significantly higher plant height and fresh weight were observed in the strain DGL1-treated group compared to the control group (**Figures 2A, B**). In the 0, 5, 10, and 15% PEG treatments, the root length of alfalfa in the control group continued to increase, indicating that the low concentration of drought stress promoted the elongation and growth of the root system. The number of root tips, average diameter, root length, surface area, and volume of alfalfa increased in the strain DGL1 treatment group compared to the control group. DGL1 significantly increased the number of root tips of alfalfa by 87.51 and 78.79% under 5 and 10% PEG, respectively (**Table 1**).

**Table 1:** Root morphology determination.

Treatment	Number of root tips	Average diameter (mm)	Root length (cm)	Surface area (cm^2^)	Volumetric (cm^3^)
CK	33.67 ± 3.21 bc	0.25 ± 0.023 a	35.36 ± 2.65 e	2.37 ± 0.15 cdef	0.02 ± 0.013 b
CK+DGL1	48.33 ± 2.52 abc	0.27 ± 0.08 a	36.36 ± 1.87 e	2.60 ± 0.48 cdef	0.02 ± 0.009 b
5% PEG 6000	37.33 ± 5.77 bc	0.29 ± 0.13 a	39.75 ± 0.78 de	3.77 ± 0.61 bc	0.03 ± 0.022 b
5% PEG 6000 +DGL1	70.00 ± 8.19 a	0.37 ± 0.10 a	57.10 ± 7.30 a	6.42 ± 1.26 a	0.10 ± 0.014 a
10% PEG 6000	38.00 ± 3.00 bc	0.32 ± 0.08 a	44.82 ± 4.60 cd	3.66 ± 0.21 bcd	0.04 ± 0.015 b
10% PEG 6000+DGL1	67.67 ± 2.08 a	0.33 ± 0.13 a	48.27 ± 4.53 bc	4.58 ± 1.66 b	0.05 ± 0.044 ab
15% PEG 6000	58.33 ± 6.43 ab	0.33 ± 0.13 a	45.53 ± 2.66 bcd	2.89 ± 1.04 cdef	0.05 ± 0.049 ab
15% PEG 6000+DGL1	68.00 ± 14.51 a	0.36 ± 0.15 a	52.16 ± 0.54 ab	3.84 ± 0.61 bc	0.05 ± 0.035 ab
20% PEG 6000	30.00 ± 14.80 bc	0.31 ± 0.09 a	19.71 ± 2.56 fg	1.89 ± 0.67 ef	0.02 ± 0.023 b
20% PEG 6000+DGL1	57.00 ± 18.00 ab	0.33 ± 0.14 a	24.42 ± 3.88 f	2.03 ± 0.88 def	0.03 ± 0.006 b
25% PEG 6000	23.00 ± 7.00 c	0.22 ± 0.07 a	14.89 ± 3.53 g	1.03 ± 0.78 f	0.01 ± 0.006 b
25% PEG 6000 +DGL1	47.67 ± 9.50 abc	0.32 ± 0.04 a	18.84 ± 2.39fg	2.23 ± 0.17 cdef	0.02 ± 0.005 b

Data were statistically analyzed using the SPSS program. Mean values were estimated based on Duncan’s test (*P*< 0.05), and indicators are expressed as the mean ± standard deviation (x± SD). Different letters indicate a significant difference (*p* < 0.05).

#### 3.2.2 Degree of peroxidation of alfalfa cell membranes

**Figure 3:**
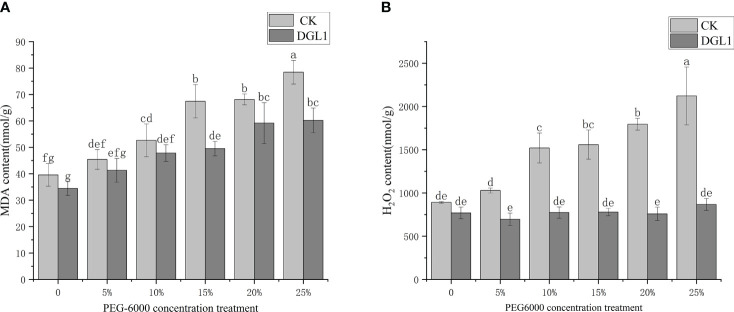
Degree of cell membrane peroxidation. **(A)** MDA; **(B)** H_2_O_2_ content. Different letters denote significant differences (P< 0.05) between various treatments based on one-way analysis of variance and Duncan’s test.

With the increase in drought stress intensity, the MDA content of alfalfa leaves in the control group showed a gradual increase and reached its maximum value at a stress intensity of 25% PEG. The MDA content of the strain DGL1 treatment group was lower than that of the control group. Under 15 and 25% PEG stress, the MDA content of the strain DGL1 treatment group significantly decreased compared to the control group. Under 5, 10, 15, 20, and 25% PEG treatments, the MDA content of inoculated DGL1 alfalfa was reduced by 9.09, 9.14, 26.52, 13.13, and 23.24%, respectively, compared to the control, indicating that DGL1 attenuated the oxidative damage caused by membrane lipid peroxidation (**Figure 3A**).

With the increase in stress intensity, the H_2_O_2_ content of alfalfa leaves in the control group showed a gradual upward trend. With a stress intensity of 25% PEG, the H_2_O_2_ content reached the maximum value, and the H_2_O_2_ content of the strain DGL1 treatment group was lower than that of the control group. Under normal water application, the H_2_O_2_ content of the strain DGL1 treatment group was lower than that of the control group, but the difference was not significant. The H_2_O_2_ content was reduced from 891.49 to 769.18 nmol/g, a decrease of 13.72%. Under 5, 10, 15, 20, and 25% PEG stress, the H_2_O_2_ content of the strain DGL1-treated group was significantly reduced compared with the control group, with decreases of 32.43, 49.16, 50.02, 57.76, and 59.15%, respectively (**Figure 3B**).

#### 3.2.3 Changes in antioxidant enzyme activities of alfalfa

**Figure 4:**
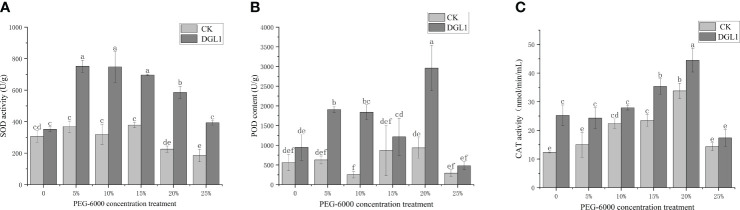
Changes in antioxidant enzyme activity. **(A)** SOD content; **(B)** POD content; **(C)** CAT content. Different letters denote significant differences (P< 0.05) between various treatments based on one-way analysis of variance and Duncan’s test.

SOD is responsible for scavenging intracellular superoxide anion radicals. With the increase in stress intensity, the SOD content of alfalfa leaves in the control group was highest at a stress intensity of 15% PEG, and the SOD content of the strain DGL1 treatment group was higher than that of the control group. Under 0, 5, 10, 15, 20, and 25% PEG treatments, the SOD content increased by 15.08, 104.16, 134.37, 84.02, 159.53, and 112.75%, respectively, compared with the control (**Figure 4A**).

POD is an important oxidative enzyme class that scavenges H_2_O_2_ from plant cells. With the increase in stress intensity, the control alfalfa leaves reached the maximum value under a stress intensity of 20% PEG. Under different stress intensity conditions, the POD content of the strain DGL1 treatment group was higher than that of the control group. Under 0, 5, 10, 15, 20, and 25% PEG treatments, the POD content of the strain DGL1 treatment group was significantly higher than that of the control group by 69.05, 204.47, 626.33, 40.00, 217.14, and 63.64%, respectively (**Figure 4B**).

CAT is an important oxidative enzyme class for scavenging H_2_O_2_ in plant cells. With the increase in stress intensity, the CAT content of alfalfa leaves in the control group showed a tendency to increase and then decrease, reaching the maximum value in the 20% PEG group. The CAT activity of the strain DGL1-treated group was higher than that of the control group, and under 5, 10, 15, 20, and 25% PEG treatments, the CAT content increased by 62.26, 24.46, 50.98, 31.68, and 21.21%, respectively, compared with the control (**Figure 4C**).

### 3.3 Effect of DGL1 on soil amelioration under drought stress

#### 3.3.1 Soil quick-acting phosphorus

**Figure 5:**
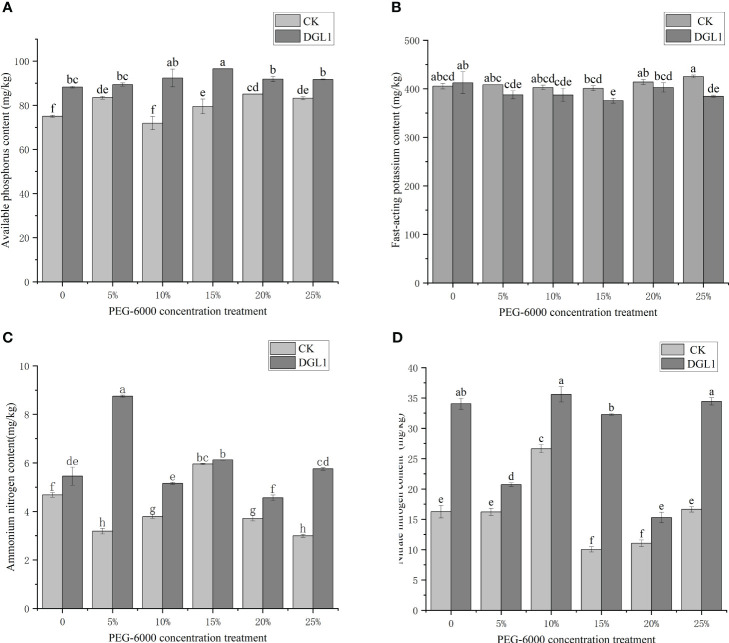
Effect of DGL1 on soil amelioration under drought stress. **(A)** Soli available phosphorus content; **(B)** Soil quick-acting potassium content; **(C)** Soil nitrate nitrogen content; **(D)** Soil ammonium nitrogen content. Different letters denote significant differences (P< 0.05) between various treatments based on one-way analysis of variance and Duncan’s test.

Under different drought stress treatments, DGL1 significantly increased the content of soil quick-acting phosphorus. Under normal water application conditions, the content of quick-acting phosphorus increased from 75.00 to 88.25 mg/kg after applying DGL1, an increase of 17.67%. Under 5, 10, 15, 20, and 25% drought stress treatments with PEG-6000, the content of quick-acting phosphorus increased by 7.11, 8.45, 21.46, 7.93, and 10.21%, respectively, compared to the control (**Figure 5A**). Based on the genome of strain DGL1, like most bacteria in the soil, the phosphate special transport system (Pst) of strain DGL1 consisted of four proteins, PstS, PstA, PstB, and PstC, and the bacteria mainly accomplished the uptake and utilization of Pi under low phosphorus stress using the Pst system. The Pst system affects bacterial phosphatase activity, accelerates the decomposition of phosphate in the environment, promotes phosphorus release, and improves phosphorus utilization in alfalfa (Martín and Liras, 2021).

#### 3.3.2 Quick-acting potassium in soil

Under drought stress, the quick-acting potassium content of the DGL1 treatment group was lower than that of the control group, and only 15 and 25% PEG treatments showed a significant decrease in quick-acting potassium content compared with that of the control group. Under normal water application, the quick-acting potassium content increased from 405.34 to 412.22 mg/kg, which was an increase of 1.70%. Under the 5, 10, 15, 20, and 25% PEG-6000 drought stress treatments, the quick-acting potassium content was reduced by 5.12, 3.87, 6.37, 2.74, and 9.56%, respectively, compared to the control (**Figure 5B**). Strain DGL1 reduced the content of quick-acting potassium in the soil. It was presumed that strain DGL1 activated the alfalfa potassium ion channels, promoted the absorption and utilization of potassium ions in the soil, and regulated cell membrane permeability as a means of maintaining the plant’s osmotic pressure. Therefore, the content of quick-acting potassium in the soil was reduced compared to that of the control.

#### 3.3.3 Soil ammonium nitrogen and nitrate nitrogen

*Bacillus* has nitrogen-fixing enzymes that can fix atmospheric nitrogen into ionic form, promote plant growth, and increase the soil nitrogen fertilizer content. Under different drought stress treatments, DGL1 significantly increased the soil nitrate–nitrogen content. Under normal water application conditions, the nitrate nitrogen content increased from 16.25 to 34.04 mg/kg, an increase of 109.48%. Under 5, 10, 15, 20, and 25% PEG drought stress treatments, the nitrate nitrogen content increased by 27.94, 33.68, 209.20, 38.37, and 106.97%, respectively, compared to the control (**Figure 5C**). Under different drought stress treatments, DGL1 significantly increased the soil ammonium nitrogen content, except with the 15% PEG treatment, which increased by 16.45% from 4.68 to 5.45 mg/kg under normal water application conditions. Under 5, 10, 15, 20, and 25% PEG drought stress treatments, the ammonium nitrogen content increased by 174.29, 36.15, 2.86, 22.91, and 92.31% (**Figure 5D**).

### 3.4 Effect of DGL1 on the metabolome of alfalfa under drought stress

#### 3.4.1 PCA analysis

**Figure 6:**
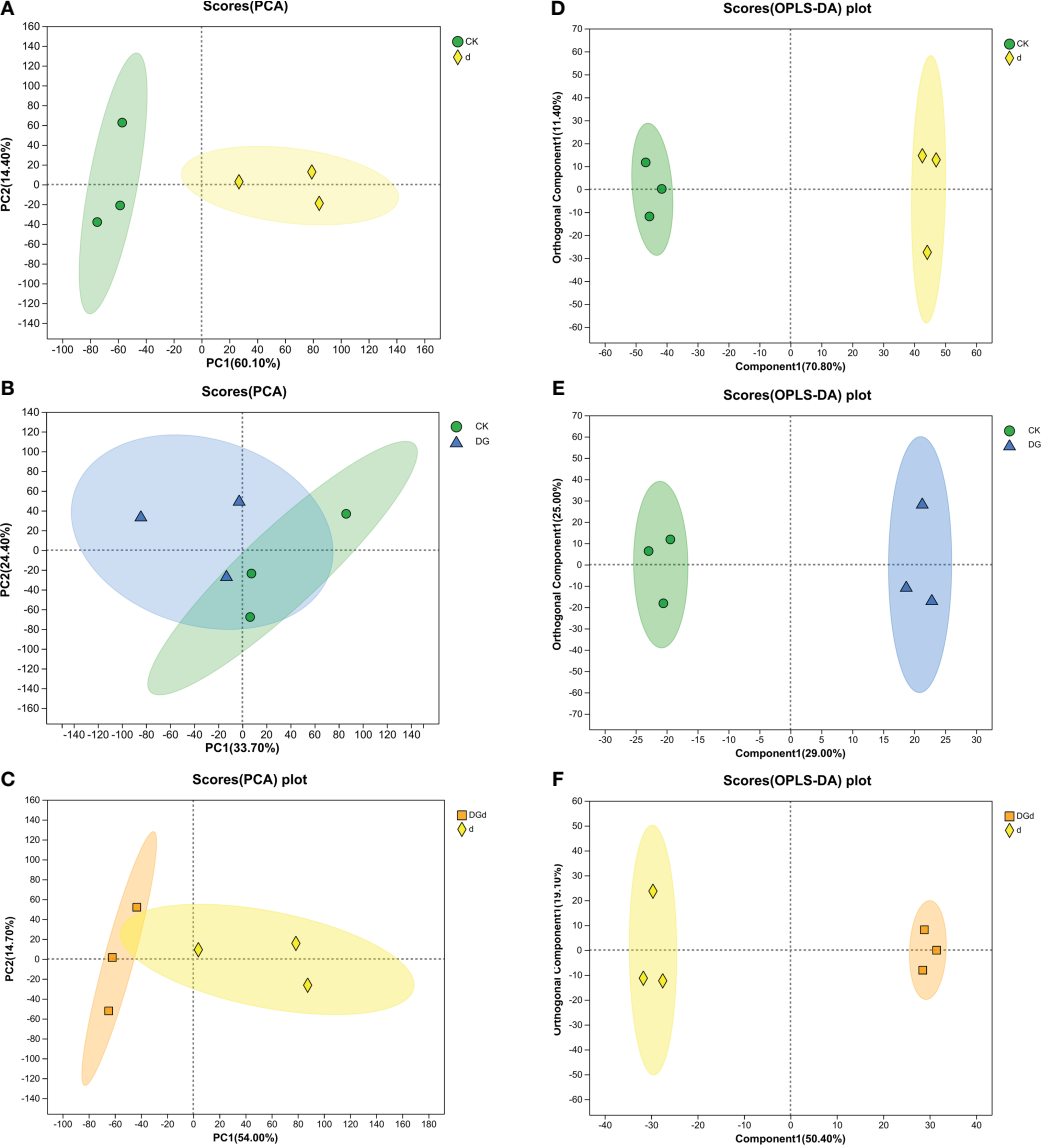
Principal component analysis (PCA) and OPLS-DA analysis. **(A–C)** PCA analyses of CK vs d, CK vs DG, and d vs DGd, respectively; **(D–F)** OPLS-DA analyses of CK vs d, CK vs DG, and d vs DGd, respectively. The distances of each coordinate point in the PCA plot represent the degree of aggregation and disaggregation among samples. The closer the distances, the greater the similarity among samples, and the further the distances, the greater the dissimilarity among samples. The greater the degree of separation between the two groups of samples in the OPLS-DA plot, the more significant the classification effect.

PCA reflects the overall metabolic differences among alfalfa samples. In this experiment, the PCA results showed that the total cumulative contributions were 54.1, 74.1, and 74.1% for the first component and 54.1, 74.5, and 68.7% for the second component in the three comparison groups “CK vs d”, “CK vs DG”, and “d vs DGd”, respectively, indicating significant differences in metabolite changes between drought-stressed strain DGL1-treated alfalfa and the control (**Figures 6A–C**).

#### 3.4.2 Orthogonal partial least squares discriminant analysis

OPLS-DA filters out information that is not relevant to classification and accurately analyzes differences between groups. The mass spectrometry data obtained were analyzed by OPLS-DA, and the three comparison groups of “CK vs d”, “DG vs CK”, and “d vs DGd” samples were distributed in different quadrants of the confidence intervals, with obvious differentiation effects, which indicated that there were significant differences between the comparison groups (**Figures 6D–F**). Its model quality parameters were as follows for the three principal components: Q_2 = _0.959, R_2_X = 0.822, and R_2_Y = 0.988 for the cumulative prediction rate of the model d vs CK; Q_2 = _0.716, R_2_X = 0.54, and R_2_Y = 0.994 for the cumulative prediction rate of the model DG vs CK; Q_2 = _0.782, R_2_X = 0.696, and R_2_Y = 0.997 for the cumulative prediction rate of the model d vs DGd. Q_2_ values > 0.5 for each group indicate that the model has good predictive ability.

#### 3.4.3 Analysis of differential metabolites between groups

**Figure 7:**
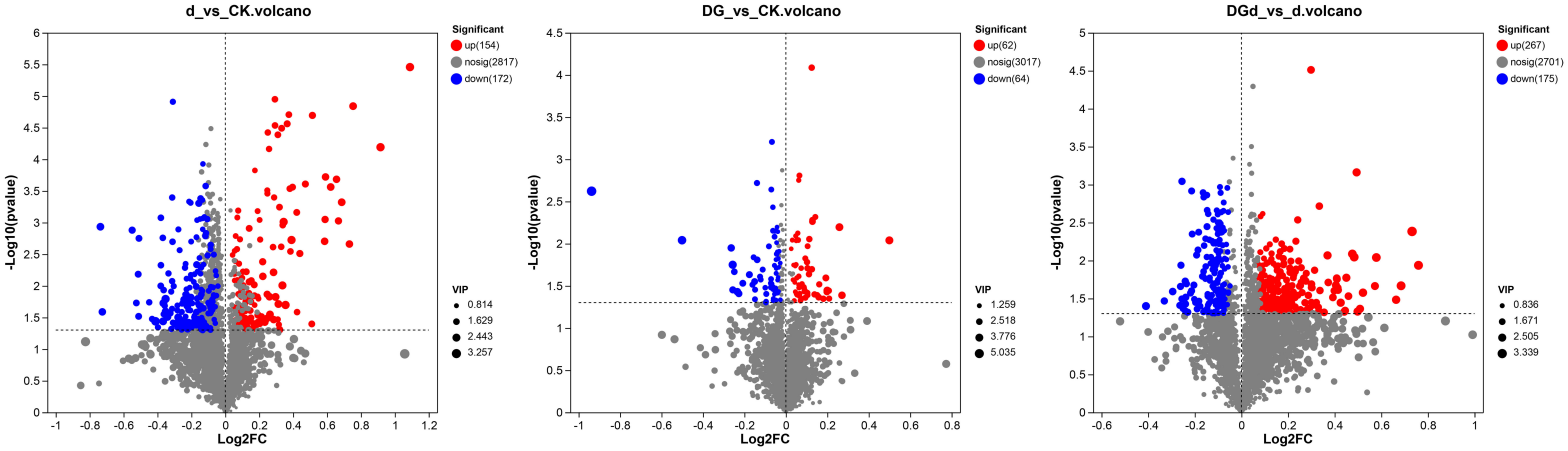
Volcano plot of differential metabolites. The horizontal coordinate is the value of log_2_FC for the fold change of the difference in metabolite expression between the two groups, and the vertical coordinate is the value of the statistical test for the difference in the change in metabolite expression −log10 (*P* value), with higher values indicating more significant differences in expression.

Metabolomics analysis revealed that 126 metabolites were changed in strain DGL1-treated alfalfa compared with the control group (DG vs CK). In positive ion mode, there were 64 differential metabolites, of which 27 were upregulated and 37 were downregulated. In negative ion mode, there were 62 differential metabolites, of which 35 were upregulated and 27 were downregulated (**Figure 7A**). A total of 326 metabolites were altered in alfalfa under drought stress compared with the control (d vs CK), including 148 differential metabolites in positive ion mode (46 upregulated and 102 downregulated) and 178 differential metabolites in negative ion mode (108 upregulated and 70 downregulated) (**Figure 7B**). A total of 442 metabolites were altered in alfalfa treated with strain DGL1 under drought stress compared with the drought stress-treated group (DGd vs d). In positive ion mode, there were 245 differential metabolites, of which 187 were upregulated and 58 were downregulated; in negative ion mode, there were 197 differential metabolites, of which 80 were upregulated and 117 were downregulated (**Figure 7C**). Analysis of the data revealed that the differential metabolites were mainly dominated by amino acids and their derivatives and lipids.

#### 3.4.4 KEGG analysis of differential metabolites

**Figure 8:**
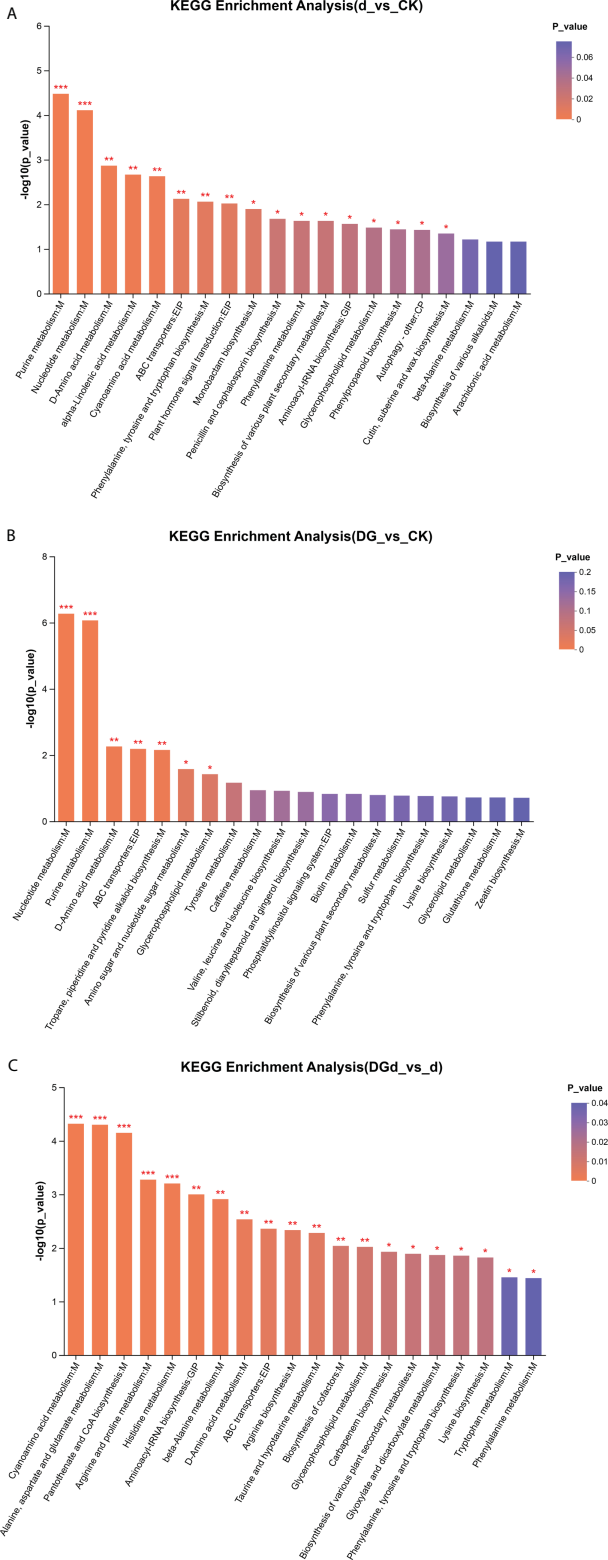
KEGG enrichment map of differential metabolites. **(A–C)** are d vs CK, DG vs CK, and DGd vs d, respectively. The horizontal coordinate is the KEGG pathway, and the vertical coordinate is the statistical test value of the difference in metabolite expression change −log10 (p-value) value. The higher the value, the more significant the difference , and Pvalue < 0.001 is labeled ***, Pvalue < 0.01 is labeled **, and Pvalue < 0.05 is labeled *.

Enrichment analysis of differential metabolites was performed with default settings using the BH method to correct for *P* values, and when the corrected *P* value was< 0.05, the pathway was considered to be significantly enriched. Differential metabolites were significantly enriched in nucleotide metabolism, purine metabolism, D-amino acid metabolism, ABC transporter proteins, tropane, piperidine, and pyridine alkaloid biosynthesis, amino acid and nucleotide metabolism, glycerophospholipid metabolism, tyrosine metabolism, caffeine metabolism, and biosynthesis of valine, leucine, and isoleucine in the strain DGL1-treated alfalfa group compared to the control group (DG vs. CK) (**Figure 8A**). Differential metabolites were significantly enriched in purine metabolism, nucleotide metabolism, D-amino acid metabolism, alpha-linolenic acid metabolism, cyanoamino acid metabolism, ABC transporter proteins, biosynthesis of phenylalanine, tyrosine, and tryptophan, phytohormone signaling, monobactam biosynthesis, biosynthesis of penicillin and cephalosporin, phenylalanine metabolism, and biosynthesis of various plant secondary metabolites compared to the control group under drought stress in alfalfa (d vs CK) (**Figure 8B**). Differential metabolites were significantly enriched in cyanoamino acid metabolism, alanine, aspartate, and glutamate metabolism, pantothenic acid and coenzyme A biosynthesis, arginine and proline metabolism, histidine metabolism, β-alanine metabolism, aminotransferase-tRNA biosynthesis, D-alanine metabolism, ABC transporter proteins, arginine biosynthesis, taurine, and hypotaurine metabolism in alfalfa treated with strain DGL1 under drought stress compared to the drought stress treatment group (DGd vs d) (**Figure 8C**).

#### 3.4.5 Comparison of GC-TOF-MS non-targeted differential metabolites between samples

GC-MS analysis showed that strain DGL1 treatment resulted in significant differences in metabolites in alfalfa compared with the control (DG vs CK). The differential metabolites in alfalfa plant tissues were mainly dominated by organic acids and derivatives, lipids and lipid-like molecules, organic heterocyclic compounds, organo-oxygenated compounds, and phenylpropanoid and polyketide metabolites. In the organic acid group, metabolites of histidine, isoleucine, phenylalanine, citrulline, tyrosine, ornithine, glutamate, and gluconic acid were significantly increased in the alfalfa leaves after DGL1 application.

**Figure 9:**
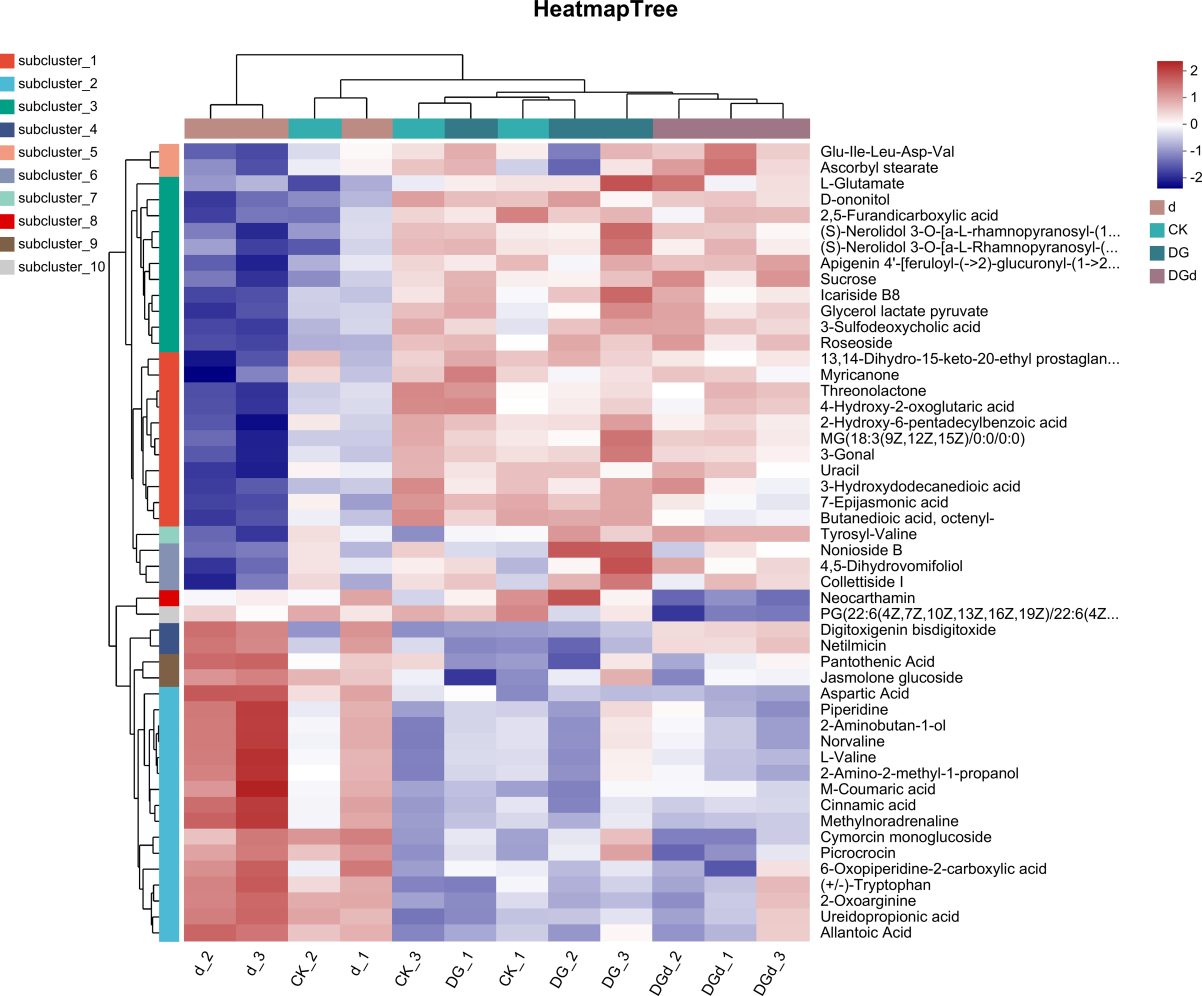
Heat map of metabolite enrichment. The left side is the dendrogram of metabolite clustering, and the right side is the name of the metabolites. The closer the two metabolite branches, the closer their expression. The upper side is the dendrogram of sample clustering, and the lower side is the name of the samples. The closer the two sample branches, the closer the expression patterns of all metabolites of the two samples, i.e., the closer the change trend of metabolite expression.

Differential metabolites in the alfalfa drought stress treatment group compared to the control (d vs CK) were mainly categorized as metabolites of lipids and lipid-like molecules, organic acids and their extensions, organic heterocyclic compounds, organic oxides, phenylpropanoids, and polyketides. In the group of lipids and lipid-like molecules, the metabolites of isopropyl glycol, cinnamon glucoside, and 3-nonenol acetate were significantly downregulated, and significant downregulation of histidine related to organic nitrogen compounds, lignans, neo-lignans, and related compounds occurred. Differential metabolites were significantly enriched in lipids and lipid-like molecules, organic acids and their derivatives, organic heterocyclic compounds, organic oxygen compounds, phenylpropanoids, and polyketides, and benzene compound-related metabolites in the alfalfa treatment group under drought stress when DGL1 was applied compared to the drought stress treatment group (DGd vs d). The sugar metabolites of alfalfa treated with DGL1 under drought stress underwent differential changes. Polysaccharides, including alginate, sucrose, and gentianose, increased after DGL1 induction, and the amino acids arginine, leucine, glutamic acid, and tyrosine also increased significantly (**Figure 9**).

## 4 Discussion

Plants subjected to a certain degree of drought stress can have metabolic disorders that adversely affect plant morphology and physiology. *Bacillus* has been reported to enhance plant tolerance through several pathways, such as producing ACC deaminase and extracellular polysaccharide EPS, increasing plant antioxidant enzyme activity, and improving soil physicochemical properties, thus increasing the biomass of the plant under stress (Gagné-Bourque et al., 2016). *Bacillus subtilis* B26 can successfully colonize the internal tissues of *Phleum pratense* under drought stress, resulting in a significant increase in aboveground and root biomass by 26.6 and 63.8%, respectively, and positively affecting plant growth and development compared to non-inoculated plants (Li and Wang, 2002). In this study, the effect of DGL1 on the growth of alfalfa under drought stress was determined, and strain DGL1 significantly increased the root length and fresh weight, number of root tips, average diameter, root length, surface area, and volume of alfalfa, indicating that strain DGL1 promoted alfalfa growth under drought stress at all stress intensities.

SOD, POD, and CAT are protective enzymes present in different locations of plant cells that synergistically scavenge reactive oxygen species and other peroxide radicals from the organism and mitigate damage to biomolecules, such as nucleic acids, proteins, and lipids (Jiaying et al., 2022). In this study, the H_2_O_2_ and MDA content increased with the enhancement of drought stress in alfalfa, indicating that drought stress causes different degrees of damage to the cell membrane system of alfalfa. Inoculation with DGL1 under drought stress resulted in a significant decrease in the MDA and H_2_O_2_ content and a significant increase in the activities of antioxidant enzymes SOD and POD in alfalfa. This suggests that DGL1 can reduce the degree of membrane lipid peroxidation in alfalfa, scavenge free oxygen radicals, and improve the redox stabilization state of plants, which, in turn, improves the adaptability of alfalfa to drought.

Phosphorus plays an important role in plant growth, development, and metabolism, while most phosphorus in the soil exists in the form of insoluble phosphate, which is difficult for plants to absorb directly and utilize (Kettenburg et al., 2023). Phosphorus-solubilizing *Bacillus* can convert phosphate into soluble phosphorus and promote soil phosphorus availability (Wang et al., 2022). In this study, strain DGL1 was found to have a phosphorus-solubilizing circle, and the amount of phosphorus solubilized by DGL1 was 44.47 ± 2.19 mg/L as determined by the vanadium-molybdenum colorimetric method. This indicates that strain DGL1 has a strong ability to solubilize phosphorus. Bacteria accomplish the uptake and utilization of inorganic phosphate mainly through the Pst system. Genomic analysis revealed that the Pst system of strain DGL1 consisted of four proteins, PstS, PstA, PstB, and PstC, which have been reported to accelerate the decomposition of phosphorus-containing organic compounds in the environment by influencing the activity of phosphatase, promoting the release of phosphorus, and increasing the utilization of phosphorus by plants (Qi et al., 1997). In this experiment, the content of quick-acting phosphorus in soil treated with DGL1 was significantly higher. Therefore, DGL1 increased the content of effective phosphorus in the soil under drought and promoted the uptake and utilization of phosphorus by the plant under drought stress, thus enhancing the drought tolerance of alfalfa.

The genus *Bacillus*, with its diverse physiological characteristics, produces a variety of antimicrobial agents and enzymes, as well as the enzyme ACC deaminase, which can degrade the synthetic precursor of ethylene, ACC, into α-Ketobutyric acid and ammonia. It not only reduces the accumulation of ethylene in plants under stress but also provides a nitrogen source for the host plant, which enhances the drought tolerance of plants, such as *Zea mays*, *Pisum sativum*, and *Triticum aestivum*, and mitigates drought damage to plants (Zahir et al., 2009; Yuan et al., 2023). In this study, strain DGL1 was found to have ACC deaminase-producing activity, and the deaminase activity was 25.25 ng/L. Therefore, it was hypothesized that strain DGL1 promoted drought tolerance in alfalfa by secreting ACC deaminase. Some bacteria can maintain a stable combination of soil and water by producing EPS, which increases the soil water content and water utilization by plants (Voběrková et al., 2016). Strain DGL1 was found to have EPS-producing activity, and genome analysis revealed that it contained *malL*, *melA*, *bglA*, *xynD*, *srfJ*, *csn*, and other genes related to EPS synthesis. Thus, strain DGL1 may increase the water content of the soil through EPS and improve the alfalfa yield under drought stress.

OPLS-DA analysis showed that the Q2 value of each metabolic group was > 0.5, indicating that the predictive ability of the model was good and could be used for subsequent analysis. Lipids are not only components of biological membranes but also play important roles in signal transduction, energy supply, and stress response. Plants can respond to adversity stress by altering the content and composition of lipids and lipid-like substances. Changes in the content of substances in the membrane lipids of *Armeniaca vulgaris* leaves in response to drought stress have been reported, with a decrease in the linolenic acid content and an increase in the stearic, oleic, and linoleic acid content in response to drought stress (Ping and Rui et al., 2002). Using metabolomic analysis, strain DGL1 treatment was shown to result in the production of organic acids and derivatives, lipids and lipid-like molecules, organic heterocyclic compounds, organic oxides, benzene-related metabolites, phenylpropanes, and polyketide metabolites in alfalfa. Among the three comparison groups, the highest percentage of lipid and lipidoid content was detected in the leaves of alfalfa, with significant differential changes in the content of lipids and lipidoids in the strain DGL1-treated group (DG) and the strain DGL1+drought stress group (DGd), suggesting that alfalfa lipids and lipidoids may play a role in plant resilience under DGL1 treatment. Benzene compounds are a specific class of aromatic metabolites, most of which are present in plants as volatile organic compounds (VOCs), and VOCs can withstand harsh environments and participate in direct and indirect plant defense (Brosset et al., 2022). We found that the content of polysaccharides, including trehalose, sucrose, and gentianose, was also significantly upregulated under drought with the application of DGL1. Trehalose, as an osmotic regulator, effectively maintains biofilm stability and intracellular active substances under stress conditions and improves a plant’s ability to tolerate abiotic stress, such as drought. Sucrose is a product of plant photosynthesis, providing energy and carbon sources for plant growth and metabolism, and it is also an important signaling regulator of gene expression (Tong et al., 2022). Amino acids, as the basic components of proteins, not only play a role in a variety of physiological functions in various stages of plant growth and development but are also key components involved in the synthesis of secondary metabolites related to plant defense against adversity stress (Dinkeloo et al., 2018). For example, tyrosine and proline can improve drought tolerance in plants, arginine can enhance root system development, and phenylalanine can promote lignin synthesis, which in turn can help the plant achieve a high yield (Tegeder, 2012). In this study, the content of arginine, leucine, glutamic acid, and tyrosine showed an upregulation trend under drought stress in the DGL1-applied group compared with the drought group. Similarly, histidine, isoleucine, phenylalanine, citrulline, tyrosine, ornithine, and glutamic acid showed an upregulation trend in the DGL1-applied group compared with the control group. DGL1 may affect the processes of amino acid metabolism, polysaccharide metabolism, and lipid metabolism in alfalfa, enhancing the capacity of the plant to resist drought stress. *Bacillus* can not only directly promote plant growth but also improve the soil rhizosphere microecological environment and soil fertility, thus indirectly promoting plant growth (Wang et al., 2020). Irrigating the root system of alfalfa with DGL1 increased the content of quick-acting phosphorus, quick-acting potassium, ammonium nitrogen, and nitrate nitrogen in the soil under drought stress, thus suggesting that DGL1 has a better ameliorating effect on the soil and increases the uptake of nitrogen, phosphorus, and potassium by alfalfa under drought stress.

## 5 Conclusion

**Figure 10:**
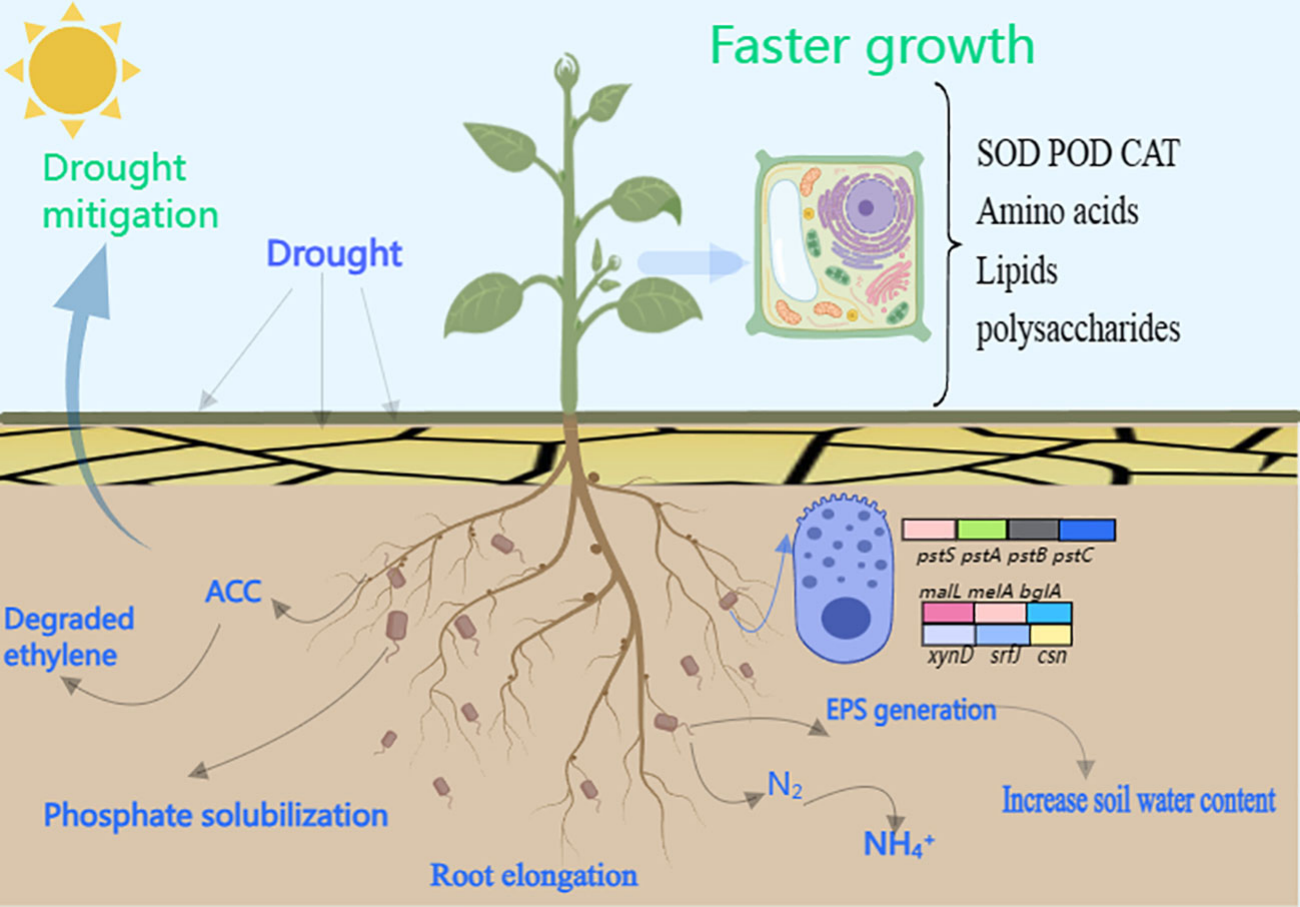
Effect of alfalfa inoculation with DGL1.

In this study, it was found that DGL1 promoted the biomass of alfalfa under drought stress, significantly increased the activities of antioxidant enzymes SOD, POD, and CAT, and decreased the MDA and H_2_O_2_ content in alfalfa under drought stress compared with the control group. DGL1 improved the adaptability of alfalfa to drought through ACC deaminase and EPS production and increased the content of quick-acting phosphorus, quick-acting potassium, ammonium nitrogen, and nitrate nitrogen in soil under drought stress, suggesting that DGL1 has an obvious ameliorating effect on the soil. Through metabolomics analysis, DGL1 was shown to affect amino acid metabolic pathways, such as arginine, leucine, glutamate, and tyrosine, as well as the levels of energy-providing polysaccharides and lipids, in alfalfa under drought stress. Thus, strain DGL1 integrated several factors to enhance the drought suitability of alpine forage alfalfa, demonstrating the potential of DGL1 for the development of microbial preparations for use in dry zones (**Figure 10**).

## Data availability statement

The original contributions presented in the study are included in the article/supplementary material. Further inquiries can be directed to the corresponding author.

## Author contributions

XY: Conceptualization, Data curation, Methodology, Writing – original draft, Writing – review & editing. YX: Funding acquisition, Project administration, Resources, Writing – review & editing. YQ: Resources, Writing – review & editing. FC: Supervision, Writing – review & editing. TW: Supervision, Writing – review & editing. JL: Supervision, Writing – review & editing. LW: Supervision, Writing – review & editing. CL: Supervision, Writing – review & editing. YG: Supervision, Writing – review & editing.
